# Cell Fractionation and the Identification of Host Proteins Involved in Plant–Virus Interactions

**DOI:** 10.3390/pathogens13010053

**Published:** 2024-01-05

**Authors:** Amany E. Gomaa, Kaoutar El Mounadi, Eric Parperides, Hernan Garcia-Ruiz

**Affiliations:** 1 Department of Plant Pathology and Nebraska Center for Virology, University of Nebraska-Lincoln, Lincoln, NE 68583, USAeparperides2@huskers.unl.edu (E.P.); 2 Department of Botany, Faculty of Science, Mansoura University, Mansoura 35516, Egypt; 3 Department of Biology, Kutztown University of Pennsylvania, Kutztown, PA 19530, USA

**Keywords:** protein–protein interactions, cell fractionation, mass spectrometry, subcellular localization, virus–host interactions

## Abstract

Plant viruses depend on host cellular factors for their replication and movement. There are cellular proteins that change their localization and/or expression and have a proviral role or antiviral activity and interact with or target viral proteins. Identification of those proteins and their roles during infection is crucial for understanding plant–virus interactions and to design antiviral resistance in crops. Important host proteins have been identified using approaches such as tag-dependent immunoprecipitation or yeast two hybridization that require cloning individual proteins or the entire virus. However, the number of possible interactions between host and viral proteins is immense. Therefore, an alternative method is needed for proteome-wide identification of host proteins involved in host–virus interactions. Here, we present cell fractionation coupled with mass spectrometry as an option to identify protein–protein interactions between viruses and their hosts. This approach involves separating subcellular organelles using differential and/or gradient centrifugation from virus-free and virus-infected cells (1) followed by comparative analysis of the proteomic profiles obtained for each subcellular organelle via mass spectrometry (2). After biological validation, prospect host proteins with proviral or antiviral roles can be subject to fundamental studies in the context of basic biology to shed light on both virus replication and cellular processes. They can also be targeted via gene editing to develop virus-resistant crops.

## 1. Introduction

Plant viruses are obligate intracellular parasites that depend on their host’s biological machinery to replicate and move cell-to-cell and systemically. Plant virus genomes encode a small set of proteins that hijack a variety of cellular processes and interact with host proteins and other biological molecules [1]. In response to viral infection, a subset of host proteins change their subcellular localization and/or accumulation level, and host organelles undergo some functional and structural changes [2,3]. For any virus–host combination, host proteins may not be involved or have a role in the interaction. The latter group can be divided into proteins with antiviral activity or a proviral role. Antiviral proteins actively defend against the virus while proviral proteins support the virus in critical parts of replication or spread within the host [4,5,6]. Therefore, the susceptibility or resistance of plants to viruses is influenced by changes that occur in the host at the protein level. Identification of proviral proteins can establish the foundation to engineer antiviral resistance by eliminating or mutating genes that determine susceptibility [7,8].

Several techniques have been used to identify interaction partners and track and measure protein accumulation, subcellular localization, and biological activity during viral infection, including 2D-gel electrophoresis, protein microarrays, yeast two hybridization, affinity purification, confocal microscopy, and bimolecular fluorescence complementation [9,10,11,12]. The yeast two hybrid (Y2H) approach allows the detection of soluble protein interactions within the cellular environment. However, it can only detect about 25% of interactions as it requires preparation of a library of bait and pray cloned proteins [13]. Other methods such as coimmunoprecipitation (Co-IP), isobaric tags for relative and absolute quantification (iTRAQ), phase separation, proximity labeling [14], and protein interaction reporter technology [15] have also be used for detecting protein–protein interactions in plant–virus combinations. These methods require cloning and tagging individual viral proteins alone or in the context of an infectious virus clone. While these approaches have provided fantastic results for key viral proteins of representative or model species [10,11,16,17], most plant viruses have not been cloned. Additionally, due to strict genome constrains or possible interference with protein function, it is likely that many viruses or viral proteins will not tolerate even simple tags (FLAG, myC, HA, HIS, etc.), as shown for *Pea enation mosaic virus 1* (PEMV1) where the addition of 3xHA to the coat protein significantly reduced PEMV1 accumulation [18]. Here, we propose the combination of tag- and clone-independent subcellular fractionation and mass spectrometry to identify, at the proteome level, host proteins interacting with viral proteins.

## 2. Cellular Organelles Are Involved in Virus Infection

Viruses have evolved to take advantage of host organelles to form their replication compartments and replicate their genomes with help from cellular proteins [19]. This section highlights some examples of organelle involvement in virus replication. Upon infection with *Tomato mosaic virus* (ToMV), 130 KDa and 180 KDa replication proteins associate with intracellular membranes and bind to host proteins such as TOM1. This binding is required for RNA-dependent RNA polymerase (RdRp) activity and ToMV genome replication [20,21].

The host secretory pathway is used for replication by some viruses. During infection with *Tobacco mosaic virus* (TMV), the host endoplasmic reticulum (ER) is transformed into large irregular aggregates used to form virus replication compartments that contain viral replication proteins, viral genomic RNA, and other host factors including ER resident chaperones such as BiP, PDI, and SKP1 [22,23,24]. The ER also plays a role in viral transport through cells, and some viruses such as TMV modify the ER tubules or form motile vesicles detached from the ER that are then targeted to endosomes [25].

Chloroplasts play a pivotal role for some plant viruses. The chloroplast unusual positioning protein 1 (CHUP1) facilitates the movement of *Cauliflower mosaic virus* (CaMV) and its inclusion bodies between cells through interaction with virus protein P6. Accordingly, silencing CHUP1 delays viral movement [26,27]. Moreover, chloroplast membranes serve as platforms for *Potexvirus* replication, with viral proteins including ribonucleoprotein complex (RNP) associating with host factors such as chloroplast phosphoglycerate kinase (chlPGK) and chaperonin Hsp90 to form viral replication compartments [28,29]

The chloroplast, Golgi apparatus, and ER are involved during infection by *Turnip mosaic virus* (TuMV). Upon infection, these organelles amalgamate into a perinuclear globular structure within which viral replication takes place [30].

Extracellular vesicles have dual roles in viral infection. In TuMV-infected plants, extracellular vesicles carry host proteins needed for virus replication proteins (poly(A)-binding protein) and antiviral defense proteins (AGO2 and 14-3-3 protein), potentially serving both as viral movement mediators and immune signal transmitters [31].

Other organelles such as mitochondria and the nucleolus are also involved in viral infection. The mitochondria participate in electron transport chain. Infection by *Cucumber mosaic virus* (CMV) disrupts electron transport chains in chloroplasts and mitochondria, leading to higher electron flow to oxygen (O_2_) and increased accumulation of hydrogen peroxide (H_2_O_2_) [32]. The nucleolus is targeted by some viral proteins such as the 3a movement protein encoded by CMV, protein P3 of *Tobacco etch virus*, and the coat protein (CP) of *Tomato yellow leaf curl virus*, which serves as a nuclear shuttle to facilitate the transport of viral DNA into and out of the nucleus, where replication takes place [33]. During infection with *Groundnut rosette virus* (GRV), the long-distance movement protein (MP) associates with host fibrillarin and travels to the nucleolus. Thus, there is a connection between fibrillarin binding and nucleolar trafficking in plant–virus interactions [34,35].

Great progress has been made in understanding interactions between viral proteins and cellular components using various techniques, such as proteomics, transcriptomics, and advanced imaging technologies [10,15,16,17]. However, there are still significant gaps in our understanding of the repertoire of interactions between plant viral proteins and cellular components. The identity of host factors, their mechanistic role, and their contribution to antiviral defense or viral replication and pathogenesis require further investigation.

## 3. Host Proteins Participate in Virus Infection

Plant host proteins play several contrasting roles during viral infection, such as being proviral or antiviral. These roles influence the progression of the disease and the outcome of the interaction. Proviral proteins are host factors that viruses exploit for their replication [36,37], cellular movement [38], or for overcoming antiviral defenses [8]. Potyviruses selectively require different eIF4E paralogs to translate their proteins and recruit them through protein–protein interactions using potyviral VPg [39]. Thus, the translation initiation factor eIF(iso)4E is a proviral host protein. Plant susceptibility to *Tobacco etch virus* (TEV) and TuMV depends on the presence of eIF(iso)4E. Mutant *Arabidopsis thaliana* plants lacking eIF(iso)4E are resistant to TEV and TuMV [40]. Similarly, reducing the expression of eIF(iso)4E in plum plants confers resistance to *Plum pox virus* [41].

Host proteins with antiviral activity are involved in various defense pathways such as RNA silencing, the hypersensitive response, autophagy, RNA decay, or pathogenesis-related proteins [42,43,44]. RNA-binding proteins, such as PUMILIO proteins bind to target RNAs using specialized RNA-binding domains and can directly or indirectly contribute to the plant defense system against RNA viruses [6,45]. Components of RNA silencing such as Dicer-like (DCL) proteins, Argonaute (AGO) proteins, and RNA-dependent RNA polymerases (RDRs) have antiviral roles [46,47].

## 4. Changes in Accumulation and Subcellular Localization of Host Proteins

**Figure 1:**
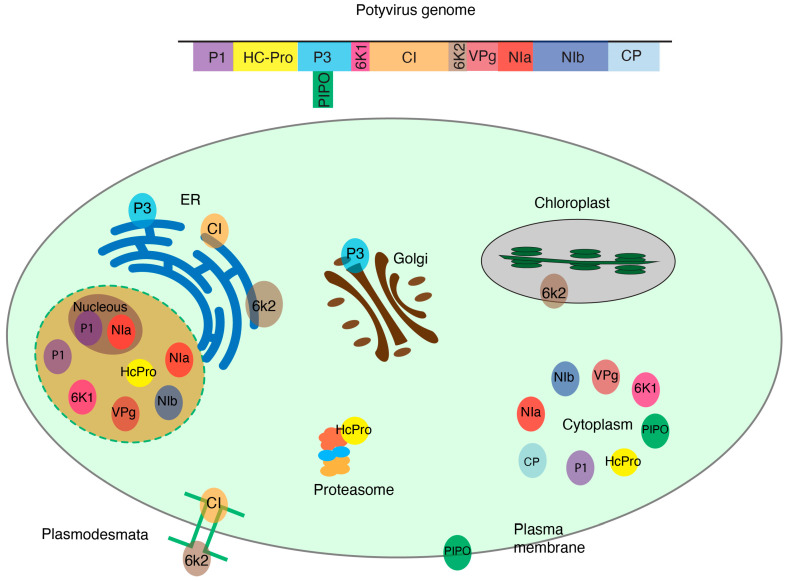
Subcellular distribution of potyviral proteins withing plant cell organelles. Potyviruses encode 11 proteins that localize to specific subcellular compartments where they interact with host proteins. Cellular proteins may change their accumulation or localization during virus infection.

**Figure 2:**
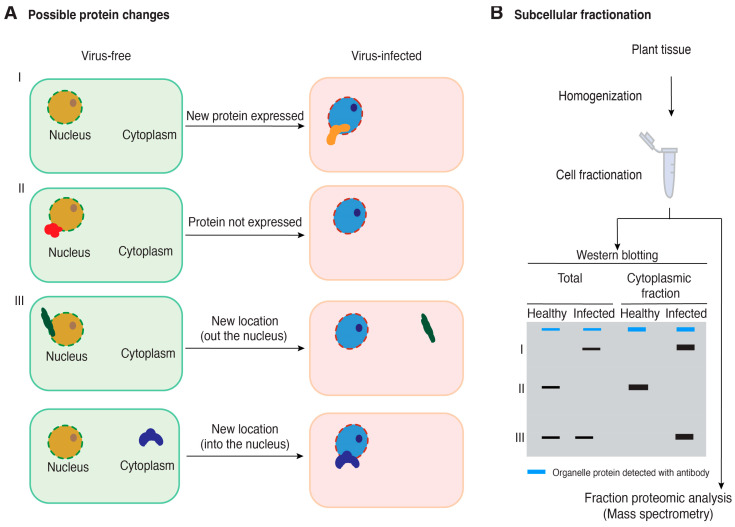
A model for the possible changes in host protein accumulation and subcellular localization during virus infection and the use of cell fractionation coupled with mass spectrometry to identify proteins relevant to plant–virus interactions. (**A**) Possible changes in host protein accumulation and subcellular localization in virus-infected plants relative to healthy plants. (**B**) Cell fractionation and Western blotting for quality control before performing mass spectrometry to identify cellular proteins that change accumulation or subcellular localization in virus-infected plants. A blue band represents a cellular protein used as a marker to be detected using an antibody. Black bands represent hypothetical changes in accumulation and localization of host proteins for which antibodies are not available.

Viral proteins localize to particular subcellular organelles (Figure 1). A multitude of changes occur in host proteins during viral infection including changes in protein expression, accumulation and/or subcellular distribution (Figure 2). These alterations are part of the plant response to restrict virus infection or are orchestrated by the virus to create a favorable environment for its replication, movement, or antagonization of the host defense response. Interestingly, proteins involved in the interactions partially overlap between resistant and susceptible cultivars [12]. For instance, RDR1 is a critical component of antiviral defense and is activated by virus infection [48,49]. During infection with *Turnip crinkle virus,* the expression of mitochondrial matrix HSP70 and CPN60 proteins increases in infected plants as a stress response to mitochondrial damage caused by the virus [50]. In response to TMV infection, the abundance of callose synthase, an enzyme involved in the synthesis of callose, increases in resistant tobacco plants. Callose is deposited inside the plasmodesmata and blocks the cell-cell movement of the virus, contributing to antiviral defense. In contrast, in susceptible plants, TMV can circumvent this barrier by increasing the activity of the host pathogenesis-related protein PR2. This leads to an increased deposition of the enzyme β-1,3 glucanase in the plasmodesmata, β-1,3 glucanase hydrolyzes callose, thereby facilitating the movement of the virus through the plasmodesmata [51,52,53].

Another possible change in proviral factors is the expression of specific proteins only during viral infection (Figure 2A) as in the case of Kunitz peptidase inhibitor-like protein (KPILP), which is not expressed in non- infected *N. benthamiana*. KPILP is involved in chloroplast retrograde signaling regulation and stimulation of intercellular transport of macromolecules and is induced by stress or expressed upon TMV infection. In plants with KPILP knocked-down TMV accumulation and intercellular movement were reduced [54].

Viral infection can also induce changes in the subcellular localization of host proteins. These changes are necessary for viral replication, cell-to-cell movement of the virus, and other steps of the viral infection. A comprehensive list of host proteins that undergo subcellular changes induced by the viral infection is reviewed in [2].

## 5. Potyviruses as a Model for Host–Viral Protein Interactions

The genus *Potyvirus* consists of positive single-stranded RNA viruses, is one of the most diverse genera, and includes species that cause significant economic losses [55]. Potyviruses encode 11 conserved proteins (VPg, P1, HcPro, P3, P3N-PIPO, 6K1, CI, 6K2, Pro, NIb, and CP). During infection, each one of the viral proteins is translocated to cellular organelles and interacts with host proteins to facilitate replication, movement, or other aspects of viral infection (Figure 1). Some host–viral protein interactions have been identified and characterized [56,57]. Most remain unexplored.

TEV protein P1 is translocated between the nucleolus and cytoplasm and interacts with the host 80s cytoplasmic ribosomes, binding to the 60s ribosomal subunits. This interaction facilitates translation of viral proteins [58]. Potyviral VPg is covalently linked to the 5′ end of the genome and binds to the host translation initiation factors eIF4E/eIF(iso)4E which is critical for viral protein synthesis [59]. TuMV VPg interacts with and degrades suppressor of gene silencing 3 (SGS3) and RDR6, both important components of the RNA silencing pathway [60]. Potyviral P3 interacts with plant eukaryotic elongation factor 1 (eEF1A) to promote the unfolded protein response and viral pathogenesis [59]. P3N-PIPO interacts with the host plasma-membrane-localized cation binding protein PCaP1, which helps in translocating the viral protein to the plasma membrane and the plasmodesmata. This, in turn, facilitates virus movement between cells [61,62].

In *Arabidopsis thaliana* plants infected with TuMV, Nlb interacts with the host RNA helicase AtRH9 which is recruited to the viral replication compartments associated with chloroplasts. The interaction with AtRH9 enhances viral replication by stimulating the RNA-dependent RNA polymerase activity of Nlb [63].

The complex network of interactions between potyviral proteins and their host proteins and organelles illustrates the interplay between the virus and its host, which impacts viral replication, movement, and plant defense responses [11,16,57]. Mapping all these interactions is necessary for a thorough understanding of plant–virus interactions and for developing strategies to design antiviral resistance in crops.

Collectively, the 167 species of potyvirus infect approximately 318 host plant species, each with a genome that encodes at least 36,795 proteins [64]. Thus, the number of possible interactions between the 11 potyviral proteins and the 36,795 host proteins is massive. It would be impossible to study these interactions on an individual basis. The task becomes even more challenging when integrating the 26 plant virus families grouping 118 genera and 1516 species of viruses.

## 6. Cellular Fractionation to Identify Host Proteins

Interactions between plant viruses and their host proteins occur within subcellular compartments (Figure 1). Thus, identification of host proteins important in plant–virus interactions can be achieved by separating cellular compartments into fractions and proteins that co-localize in each subcellular compartment identified using mass spectrometry [15,65,66]. By comparing proteomic profiles between a virus-infected and a virus-free sample, it would be possible to identify host proteins that co-localize with viral proteins in each subcellular compartment and to determine host proteins that are lost during infection in each subcellular compartment (Figure 2).

Several studies have used cell fractionation to elucidate the interaction between viral proteins and their host counterparts (Table 1). Separation of the plasma membrane was used to identify the interaction between SMALL AUXIN UP RNA (SAUR15) and the BRASSINOSTEROID-INSENSITIVE 1 (BRI1) protein in *Arabidopsis thaliana* plants. The interaction between these two proteins activates the plasma membrane’s H^+^ ATPase, which stimulates cell growth [67]. Mitochondria-rich fractions of quinoa plants infected with a mitovirus were separated via cell fractionation to analyze the impact of viral infection on the mitochondrial proteome. The analysis revealed that the infection leads to an up-regulation of proteins that modulate stress response to drought [68]. Cell fractionation was also used to elucidate the order of enzymatic activities across the Golgi apparatus. Separation of the Golgi proteins using cell fractionation, followed by mass spectrometry, revealed the presence of differences in the sequences of transmembrane amino acids across the Golgi [69].

A proteomic comparison of a resistant and a susceptible maize cultivar inoculated with *Sugarcane mosaic virus* (SCMV, genus *Potyvirus*) showed that most of the differentially expressed proteins are predicted to localize to the chloroplast [12]. Thus, separation of the chloroplast via cell-fractionation and comparison of proteomic profiles in mock-inoculated versus potyvirus-infected plants has potential to identify host and viral proteins that are important for the interaction. Similarly, for any plant–virus combination, separating nuclear and cytoplasmic fractions or soluble and membrane fractions [70] has potential to provide novel insights into the subcellular localization of viral proteins and their possible cellular interaction partners. Furthermore, proteins that re-localize within virus-infected plants have a high probability of executing a proviral role, as described for Poly(A) binding protein 2 (PABP2) and TuMV [70]. The Groundnut rosette virus open reading frame (ORF) 3 protein inters the nucleus, interacts with and re-organizes cajal bodies, and induces their fusion with the nucleolus. Nucleolar localization of the ORF3 protein is essential for the formation of viral ribonucleoprotein particles capable of intercellular movement leading to systemic infection [35]. Tobacco rattle virus (TRV) infection induces nucleolar re-distribution of coilin via a mechanism that is dependent on TRV protein 16K interacting with Poly(ADP-ribose) polymerase 1 (PAR1), a key regulator of salicylic acid mediated defense [71]. This is critical for viruses causing important diseases that have not been extensively studied such as *Maize chlorotic mottle virus*, a causal agent of maize lethal necrosis disease [72].

**Table 1:** Representative host proteins important in plant–virus interactions that were identified via cellular fractionation.

Host	Virus	Host Protein	Viral Protein	Technique	Ref.
Transgenic tobacco BY-2 cells	ToMV	Sar1, Sec61, and TOM1	130 KDa and 180 KDa replication proteins	Membrane flotation analysis and Sucrose gradient sedimentation analysis	[20]
Transgenic tobacco BY-2 cells	ToMV	Tm-1	130 K and 180 K	Differential centrifugation	[73]
*Cucumis sativus*	ToRSV	N/A	NTB	Membrane fractionation	[74]
Pea or lettuce plants	LMV	20 s Proteasome	HCPro	30% sucrose cushion and gel filtration column	[75]
*N. benthamiana*	CiLV-C	N/A	P29, P15, MP, and P24	Bimolecular Fluorescence Complementation combined with ultracentrifugation	[76]
Tomato	TYLCV	HSP70	CP	Sucrose density gradient	[77]
*N. benthamiana*	PVA	Ck2, CPIP, HSP70, and CHIP	NIb, VPg, and CP	Membrane fractionation	[78]
*N. benthamiana*	CMoV	SUMO1, SUMO2, and SCE1	ORF4	Cell wall fractionation	[79]

Viruses: citrus leprosis virus C (CiLV-C), lettuce mosaic virus (LMV), potato virus A (PVA), tomato yellow leaf curl virus (TYLCV), tomato ringspot virus (ToRSV), tomato mosaic virus (ToMV), and carrot mottle virus (CMoV). Viral proteins: nucleoside triphosphate binding (NTB), nuclear inclusion protein b (NIb), viral protein genome-linked (VPg), coat protein (CP), movement protein (MP), and movement protein (ORF4). Host proteins: These proteins were identified via cellular fractionation: small GTP-binding protein (Sar1), protein transport protein (Sec61), target Of Myb1 Membrane Trafficking Protein (TOM1), small ubiquitin-like modifier (SUMO), SUMO-conjugating enzyme (SCE1), heat shock protein 70 (HSP70), Hsc70-interacting protein (CHIP), protein kinase CK2, and CP-interacting protein (CPIP).

## 7. The Process of Cellular Fractionation

Cell fractionation involves the disruption of the cell and tissue homogenization using techniques that range from traditional grinding with a mortar and pestle to enzymatic digestion or biochemical processes [80]. Protocols for separating each subcellular compartment (Table 2) and the buffers needed (Table 3) are summarized.

Differential centrifugation is used to enrich the target organelle and eliminate other compartments and contaminants (Figure 2). The speed of centrifugation determines which cellular compartments precipitate based on their size and density. Organelles that are larger and denser, such as non-broken cells, nuclei, or chloroplasts, precipitate at lower centrifugal forces. Mitochondria, Golgi apparatus, ER, and ribosome, on the other hand, precipitate at high speed [81]. While differential centrifugation is efficient in separating organelles, its resolving power is limited. To overcome this limitation, density gradient centrifugation is employed in later stages of the process. This technique involves creating concentration gradients using substances such as sucrose or Ficoll. When the desired fraction to be purified is loaded onto the gradient, its components reach their equilibrium density, facilitating their separation [82].

**Table 2:** Organelles markers and the methods for separating plant cellular components via differential and gradient centrifugation. All centrifugation is at 4 °C unless otherwise mentioned.

Organelle	Marker Proteins	Tissue Type	Homogeniza-tion Buffer	Centrifugation Speed, Condition	Fraction Obtained	Gradient Centrifugation	Purification	Final Obtained Fraction	Ref.
Membrane fraction	See other organelles	*Cucumis sativus* leaves	Homogenization buffer one	30,000× *g*, 30 min	Crude membrane (P30) (pellet)	20–45% sucrose gradient centrifugate at 143,000× *g* for 4 h	-	Membranes separated into 13 fractions	[74]
Cytoplasm	UDP-glucose pyrophosphorylase (UGPase) [83]	Tomato leaves	Nuclear extraction buffer	Filtrate centrifuged at 1300× *g*, 10 min	Cytoplasmic fraction concentrated 10 times by ultracentrifugation	10–50% sucrose gradient centrifugation at 104,000× *g* for 20 h	-	10 fractions obtained from the gradient	[77]
		Rice cell culture	Enzyme buffer	100,000× *g*, 1 h	Remove top lipid layer, take supernatant	-	Add trichloroacetic acid to supernatant; centrifuge at 20,000× *g* for 5 min	Wash the pellet with cold acetone at 20,000× *g* for 15 min and take the pellet	[84]
Vacuole	TIPs (α and γ isoforms), Epsilon subunit of tonoplast H+ ATPase (V-ATPase) [85] and [86]	*Arabidopsis* Rosette leaves	Protoplast solution	80× *g* at 20 °C, 15 min	Pellet protoplast	10% Ficoll buffer overlayed on 4% Ficoll and vacuole buffer; centrifugation at 50,000× *g*, 5 min at 10 °C	-	Vacuoles found on the 4% Ficoll buffer/vacuole buffer interface	[87]
Chloroplast	Plastocyanin (PC) light harvesting complex b (LHC) [83,85]	*N. benthamiana* leaves	Enzyme mixture	300× *g*, 16 min	Resuspend the pellet	40% and 80% Percoll gradient and centrifugate at 3000× *g*, 25 min; collect chloroplasts at the interface of 40%/80% Percoll	-	Resuspend chloroplast in resuspension buffer	[83]
ER	HDEL domain [85]	Castor Bean Endosperm	Homogenization buffer two	1000× *g*, 15 min	supernatant	20%, 30%, 40%, and 60% sucrose; centrifuge at 250,000× *g*, 22 h at 2 °C	Resuspend ER fraction between 20% and 30% and pellet via centrifugation at 250,000× *g* for 45 min	Resuspend ER pellet	[88]
Mitochondria	Voltage-dependent, anion-selective channel protein 1-5 (VDAC1-5) [89]	Citrus pulp	Extraction buffer for mitochondria	3000× *g*, 10 min then centrifuge the supernatant at 12,000× *g*, 15 min	Resuspend the pellet in washing buffer	18%, 22.5%, and 35% Percoll gradient; ultracentrifugation at 50,000× *g*, 1 h	Mitochondrial band enriched at 22.5–35% Percoll gradient interface, then diluted with washing buffer, and centrifuged at 1500× *g*	Resuspend, purified mitochondria pellet in small volume of washing buffer	[90]
Golgi	ADP-ribosylation factor 1 (ARF1) [91]	Wheat seedling	Extraction buffer for Golgi membranes	3000× *g*, 20 min	supernatant	25–40% sucrose gradient centrifuge at 100,000× *g* for 16 h	Ultracentrifuge fractions (1:10) at 100,000× *g* for 1 h	Resuspend the membrane pellet in 50 μL dilution buffer	[92]
Nucleus	Histone H3 [93]	Tomato leaves	Nuclear extraction buffer	1300× *g*, 10 min	Pellet	10–50% sucrose gradient centrifuge at 104,000× *g*, 20 h	-	10 Nuclei fractions	[77]
Proteasomes	Regulatory Particle Triple-A ATPase subunit 2 (RPT2) and Regulatory Particle Non-ATPase 10 (RPN10) [94]	*Arabidopsis* seedlings	Extraction buffer for proteasomes	30,000× *g*, 15 min	Supernatant	Precipitation with 2% and 10% PEG 8000 then re-clarifying via centrifugation at 30,000× *g*, 45 min	Anion exchange chromatography column	Precipitation with 10% PEG 8000 then size elution chromatography to obtain peak fraction.	[95]
Plasma membrane	P-type H″-ATPase [85]	*Arabidopsis* seedlings	Homogenizing medium	2770× *g*, 10 min, take supernatant; ultracentrifugation at 231,000× *g*, 35 min	Pellet	-	Multiple ultracentrifugation	Resuspend the pellet in PM-suspension medium	[96]
Peroxisome	Catalase [97]	*Arabidopsis* rosette leaves	Grinding buffer	5000× *g*, 1 min	Supernatant free from chloroplast and nuclei	15–38% (*v*/*v*) Percoll gradient; centrifuge at 13,000× *g*, 12 min	36% sucrose centrifuge at 39,000× *g* for 30 min	Leaf peroxisome fraction located at the bottom	[98]
Autophagosome	Autophagy-related protein 8 (ATG8) [99]	Tobacco BY-2 cell suspension culture	Lysis buffer	17,000× *g*, 5 min	Pellet	30% Percoll; centrifuge at 50,000× *g*, 1 h	Place density marker beads on 30% Percoll solution and centrifuge again	Fractionate into 30 fractions	[100]
Ribosome	Ribosomal Protein S6 (RPS6) [101]	*Arabidopsis* seedlings/leaves	Ribosome extraction buffer	10,000× *g*, 15 min	Supernatant	Sucrose cushion, 149,000× *g*, 18 h	Resuspend ribosomal pellet in Staehelin A buffer; spin at 14,000× *g* for 15 min	Collect the supernatant	[102]
Extracellular vesicles	Tetraspanin 8 [103] Syntaxin PENETRATION1 (PEN1) [104]	*Arabidopsis* rosettes	Vesicle isolation buffer (VIB)	700× *g*, 20 min at 2 °C	Supernatant	-	Centrifuge successively at 10,000× *g* for 60 min, 40,000× *g* for 60 min, and 100,000× *g* for 60 min and obtain the pellet each time	Pellet resuspended in VIB	[104]

Gel electrophoresis can be used to separate protein extracts from each fraction to confirm the purity of organelle fractions during cell fractionation. The separated proteins can then be transferred onto membranes and probed using organelle markers (Table 2) that indicate the enrichment of the target organelle (Figure 2B) or the presence of contamination from non-target organelles within the fraction. This step helps in assessing the purity of the organelle fraction obtained during the fractionation process [81]. For instance, Tonoplast Intrinsic Protein (TIPs) (α and γ isoforms) and Epsilon subunit of tonoplast H+ ATPase (V-ATPase) proteins are used as markers in Western blots to confirm the extraction of the vacuole. Plastocyanin (PC) light-harvesting complex b (LHC) is used as an organelle marker for chloroplasts [83].

Following cell fractionation, mass spectrometry (MS), LC-MS, or LC-MS/MS can be used to identify and quantify the proteins present in each fraction [105]. In MS, samples are first digested using trypsin, and the peptides are then separated for quantification. In LC-MS, the MS analyses of samples are compared, while in LC-MS/MS variant a tandem mass spectrometry (MS/MS) analysis is added to provide more detailed peptide information [81]. The LC-MS approach compares the abundance of peptides in two different runs. The area under the LC-MS profile of a specific precursor ion is measured in each run. The fold change in protein abundance is then determined by comparing the areas corresponding to the same peptide in both runs. The advantage of this method is that it does not require prior peptide identification through MS/MS analysis. Peptide abundance is quantified first, and the MS/MS analysis is performed only for those peptides that show changes in abundance [106].

**Table 3:** Buffers for cellular fractionation methods.

Buffers	Components	Reference
Homogenization buffer one	50 mM Tris–HCl (pH 7.4), 15 mM MgCl_2_, 10 mM KCl, 20% glycerol, 0.1% β-mercaptoethanol, 5 μg/mL leupeptin, and 2 μg/mL aprotinin.	[107]
Nuclear extraction buffer	10 mM MES (pH 5.2), 250 mM sorbitol, 10 mM NaCl, 5 mM NaF, 5 mM EDTA, 10 mM MgCl_2_, 0.024% Triton X-100, 0.1% bovine serum albumin, 1 mM fresh DTT, and Complete Protease Inhibitor Mixture.	[77]
Enzyme buffer	0.4 M Mannitol, 3.6 mM MES–KOH (pH 5.7), 2.0% (*w*/*v*) cellulase Onozuka RS, 0.5% (*w*/*v*) pectolyase Y-23, and 1.0% (*w*/*v*) Driselase.	[84]
Protoplast solution	1% (*w*/*v*) Cellulase Onozuka R10, 1% (*w*/*v*) Macerozyme R10, 0.4 M mannitol, 25 mM CaCl_2_, 5 mM mercaptoethanol, and 10 mM 2-morpholinoethanesulfonic acid (MES)-KOH (pH 5.7).	[87]
Vacuole buffer	0.45 M mannitol and 5 mM sodium phosphate 2 mM EDTA (pH 7.5). Keep on ice. The 200 mM sodium phosphate stock solution (pH 7.5) can be prepared by mixing 84 mL of 200 mM Na_2_HPO_4_ and 16 mL of 200 mM NaH_2_PO_4_.	[87]
Enzyme mixture	1.5% (*w*/*v*) cellulase R-10, 0.5% (*w*/*v*) macerozyme R-10, 5 mM 2-morpholinoethanesulfonic acid (MES), 0.1% (*w*/*v*) BSA, 10 mM CaCl_2_, and 0.4 M mannitol (pH 5.8).	[83]
Chloroplast resuspension buffer	0.3 M sorbitol, 20 mM Tricine-KOH (pH 7.6), 5 mM MgCl_2_, 2.5 mM EDTA.	[83]
Homogenization buffer two	500 mM sucrose, 10 mM KCl, 1 mM EDTA, 1 mM MgCl_2_ 2 mM dithiothreitol (DTT), 0.1 mM phenylmethyl-sulfonyl fluoride (PMSF), and 150 mM Tricine-KOH pH 7.5.	[88]
Extraction buffer for mitochondria	0.4 M sorbitol, 0.2 M MOPS-Tris (pH 7.8), 7.5 mM EDTA, 1.5% (*w*/*v*) PVP-40, 0.1% [*w*/*v*] bovine serum albumin, and 2 mM DTT.	[90]
Washing buffer	0.33 M sorbitol and 50 mM MOPS-Tris (pH 7.5).	[90]
Extraction buffer for Golgi membranes	50 mM HEPES–KOH (pH 6.8), 0.4 M sucrose, 1 mM dithiothreitol (DTT), 5 mM MnCl2, and 5 mM MgCl_2_.	[92]
Extraction buffer for Proteasomes	50 mM potassium phosphate (pH 6), 2 mM MgCl_2_, 5% (*v*/*v*) glycerol, and 5 mM 2-mercaptoethanol supplemented with 10 mM ATP, 5% polyvinylpyrrolidone, 0.6% sodium metabisulfite, and 2 mM phenylmethylsulfonyl fluoride. 0.8% plant protease inhibitor mixture is added just before use.	[95]
Homogenizing medium	0.5 M sorbitol, 50 mM MOPS–KOH (pH 7.6), 5 mM EGTA, 5 mM EDTA, 1.5% (*w*/*v*) polyvinylpyrrolidone 40 (PVP-40, molecular weight 40,000), 0.5% (*w*/*v*) defatted-BSA, 2 mM phenylmethanesulfonyl fluoride (PMSF), 4 mM salicylhydroxamic acid (SHAM), and 2.5 mM 1,4-dithiothreitol (DTT).	[96]
Plasma membrane (PM)-suspension medium	10 mM MOPSKOH (pH 7.3), 1 mM EGTA, 0.3 M sucrose, 1 mM DTT. Store the stock solution without DTT at 4 °C.	[96]
Grinding Buffer	170 mM Tricine-KOH (pH 7.5), 1.0 M sucrose, 2 mM EDTA, 1% (*w*/*v*) BSA, 10 mM KCl, 1 mM MgCl_2_, 0.5% (*w*/*v*) PVP-40, and 5 mM DTT.	[98]
Lysis buffer	50 mM HEPES–KOH (pH 7.5) buffer containing 1 mM EDTA, 10 μM leupeptin, 10 μM pepstatin A, 1 mM AEBSF, and 0.4 M sorbitol. Mix 50 mL of 0.1 M HEPES–KOH (pH 7.5); 20 mL of 2 M sorbitol; 29 mL of water; and 1 mL of 0.1 M EDTA–NaOH (pH 8.0). Store at 4 °C. Take 10–20 mL of lysis buffer and add 1/100 volume of 1 mM leupeptin, 1/100 volume of 1 mM pepstatin A, and 1/100 volume of 0.1 M AEBSF immediately before use.	[100]
Staehelin A buffer	20 mM Tris–HCl (pH 7.5), 5 mM MgCl_2_, 1 mM sodium molybdate, and 1 mM dithiothreitol.	[102]
Ribosome extraction buffer	200 mM Tris–HCl (pH 7.5), 200 mM KCl, 25 mM EGTA, 36 mM MgCl_2_, 1 mM sodium molybdate, 1 mM dithiothreitol, 50 μg/mL cycloheximide, 50 μg/mL chloramphenicol, 80 mM β-glycerophosphate, 1% (*v*/*v*) Triton X-100, 1% (*v*/*v*) Brij 35, 1% (*v*/*v*) Tween 40, and 1% (*v*/*v*) NP40.	[102]
Vesicle isolation buffer VIB	20 mM MES, 2 mM CaCl2, and 0.1 M NaCl (pH 6).	[104]

## 8. Experimental Challenges of Cell Fractionation

Subcellular fractionation combined with mass spectrometry comes with challenges. One of them is that obtaining a pure organelle fraction might be difficult because overlapping can occur even when applying density gradient centrifugation [82]. Another is that some subcellular compartments that are challenging to fractionate or may be lost during the fractionation process. This could lead to the underrepresentation or omission of important protein–protein interactions [81]. Moreover, protein–protein interactions between plants and viruses can be highly dynamic, transient, or dependent on specific stages of infection or cellular conditions. Since cell fractionation captures protein composition at a specific time point, it may miss short-lived or context-dependent interactions that occur during specific stages of the infection [108]. Another challenge relates to the solubility of the viral and host proteins. Some membrane-associated proteins and other proteins can be difficult to solubilize or extract during fractionation, which can result in the loss or incomplete representation of these interactions in the fractionation and subsequent mass spectrometry analysis [109,110].

Proteins accumulates at variable quantities and cellular organelles vary in abundance. In published information (Table 1), the starting material was protoplasts, or 1 g to 500 g of leaf tissue. Thus, it can be predicted that the amount of tissue to start cell fractionation will also need to be adjusted for every plant–virus combination.

Mass spectrometry-based proteomics techniques also have sensitivity and dynamic range limitations that may impact the detection of low-abundance or weakly interacting proteins. They can also generate false positives and false negatives. False positives can arise from non-specific protein–protein interactions or contaminants introduced during sample processing. False negatives, on the other hand, may occur if the proteins of interest are present below the detection limit or if certain protein–protein interactions are not compatible with the fractionation or mass spectrometry techniques employed [111].

The functional relevance and significance of proteins that co-localize interactions require further validation and functional assays. Follow-up studies are necessary to understand the biological implications of the identified protein–protein interactions in the context of plant–virus interactions [112].

To overcome the limitations listed above, it is important to complement cell fractionation and mass spectrometry with other techniques, such as co-immunoprecipitation, fluorescence-based assays, or functional studies, to validate and further investigate the identified protein–protein interactions. Integrating multiple approaches can provide a more comprehensive understanding of the complex interactions between plants and viruses [113].

## 9. Conclusions

Identification and characterization of proteins that mediated host–viral interactions is critical for fundamental understanding of virus–host interactions, virus replication, cellular processes, and for developing strategies to engineer resistance to viruses in crops. Proteome-wide interaction analyses offer a promising alternative to traditional techniques, are not dependent on cloning or tagging individual proteins alone or in the context of an infectious clone, and allow for the comprehensive identification of host proteins important in host–viral interactions. To determine changes in subcellular localization and accumulation of host proteins during viral infection, we propose the following: (i) To extract proteins from a specific location in the cell in the form of purified organelle from virus-free and virus-infected plant using the methods listed in Table 1 and Table 2; (ii) Visualize proteins in the obtained fractions using sodium dodecyl sulfate-polyacrylamide gel electrophoresis (SDS-PAGE) and probe with the organelle marker antibody for quality control (Table 2); (iii) Identify and quantify proteins in each fraction using mass spectrometry; (iv) compare the changes in identity, abundance, and localization of proteins between the same fractions of virus-free and virus-infected samples.

Insights gained from studying viral–plant protein interactions have far-reaching implications for plant virology, agriculture, and our understanding of fundamental cellular processes. By unraveling the roles of host proteins in viral infection, we can potentially identify new targets for antiviral strategies, develop novel approaches to engineer resistant crops, and improve our understanding of the mechanisms governing cellular responses to viral infection.

## Data Availability

Not applicable.
